# Is countershading camouflage robust to lighting change due to weather?

**DOI:** 10.1098/rsos.170801

**Published:** 2018-02-07

**Authors:** Olivier Penacchio, P. George Lovell, Julie M. Harris

**Affiliations:** 1 School of Psychology and Neuroscience, University of St Andrews, St Andrews, Fife KY16 9JP, UK; 2 Division of Psychology, Social and Health Sciences, Abertay University, Dundee DD1 1HG, UK

**Keywords:** visual search, search efficiency, shape-from-shading, visual camouflage, countershading, foraging

## Abstract

Countershading is a pattern of coloration thought to have evolved in order to implement camouflage. By adopting a pattern of coloration that makes the surface facing towards the sun darker and the surface facing away from the sun lighter, the overall amount of light reflected off an animal can be made more uniformly bright. Countershading could hence contribute to visual camouflage by increasing background matching or reducing cues to shape. However, the usefulness of countershading is constrained by a particular pattern delivering ‘optimal’ camouflage only for very specific lighting conditions. In this study, we test the robustness of countershading camouflage to lighting change due to weather, using human participants as a ‘generic’ predator. In a simulated three-dimensional environment, we constructed an array of simple leaf-shaped items and a single ellipsoidal target ‘prey’. We set these items in two light environments: strongly directional ‘sunny’ and more diffuse ‘cloudy’. The target object was given the optimal pattern of countershading for one of these two environment types or displayed a uniform pattern. By measuring detection time and accuracy, we explored whether and how target detection depended on the match between the pattern of coloration on the target object and scene lighting. Detection times were longest when the countershading was appropriate to the illumination; incorrectly camouflaged targets were detected with a similar pattern of speed and accuracy to uniformly coloured targets. We conclude that structural changes in light environment, such as caused by differences in weather, do change the effectiveness of countershading camouflage.

## 1. Introduction

Understanding the colouring and patterning of animals is a classic problem in evolutionary biology. Animal body coloration is thought to serve several important purposes including communication, camouflage, mimicry, thermoregulation and/or protection against damaging radiation [1,2]. One example is countershading, a gradation of colour whereby the side of the body that receives the strongest illumination is darker than the opposite side [3,4]. Several theories predict the evolution of a countershading pattern, including protection from ultraviolet light and thermoregulation, but we will focus here on the hypothesis that countershading provides a form of camouflage (see [5] for a discussion of the theories' relative merits). Understanding how countershading can provide useful camouflage is complicated by the fact that the best pattern of shading is critically affected by animal shape and posture, and by lighting effects that vary with time of day and weather [6]. In this paper, we report experimental work on human observers, showing that detection of countershaded targets is affected by weather conditions that result in different optimal patterns of countershading.

**Figure 1.**
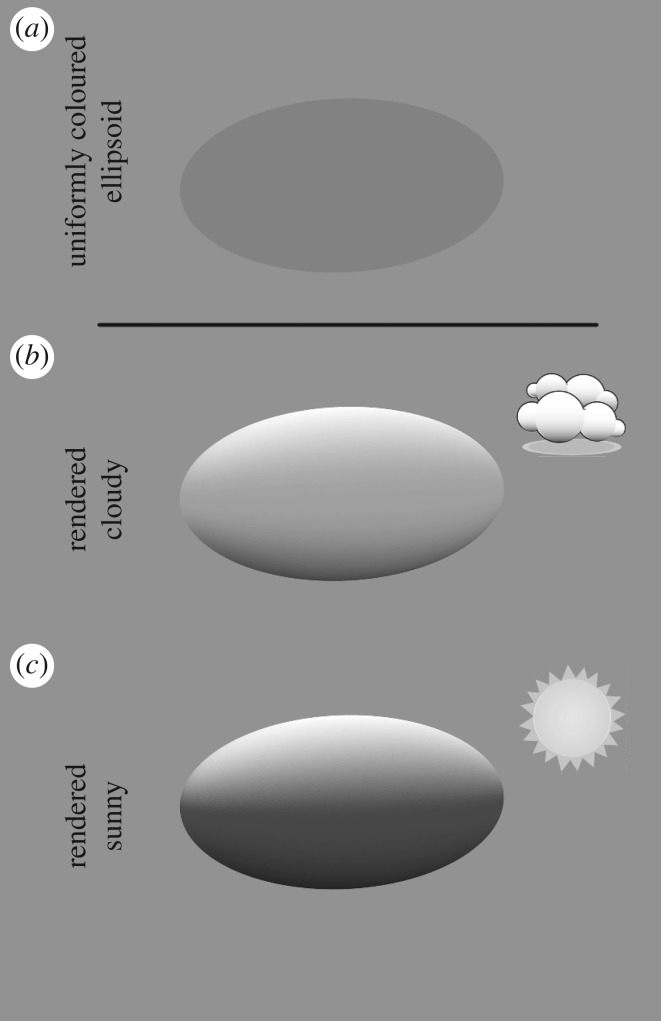
Appearance of a uniform grey-coloured ellipsoid in different lighting conditions. (*a*) Uniformly coloured grey three-dimensional ellipsoid (as seen under all-round uniform, i.e. isotropic, lighting), and its appearance under (*b*) a cloudy sky and (*c*) a sunny sky when rendered in the ray-tracing program Radiance and realistic light distributions [17].

Countershaded patterning is common across many taxa and contrasting habitats [7–13]. Explanations for the evolution of countershading typically start from the fact that in both terrestrial and aquatic environments more light comes from above. Uniformly coloured three-dimensional (3D) objects therefore appear brighter on the side that faces upwards: a phenomenon referred to as self-shadowing [3,14]. Countershading has been hypothesized to have evolved to counterbalance self-shadowing, therefore enhancing background matching and hiding information that betrays the shape of the body [3,4,7,12,14–16]. However, the degree to which brightness varies between the top surface and elsewhere on the body is dependent on the specific lighting conditions [6]. This is illustrated in figure 1, which shows how a uniformly grey ellipsoid (figure 1*a*) would appear under a cloudy sky (figure 1*b*) or a sunny sky (figure 1*c*), displaying a noticeably different pattern for each illumination.

There has been some work on how the relationship between countershading and a typical lighting environment is demonstrated in animals possessing different patterns. For example, countershaded species that live closer to the equator, or spend more time in open light environments, are associated with pelt patterns containing a stronger gradient between dark and light [7]. Using artificial prey, previous experimental studies have shown that two-tone countershading patterns, showing a darker back, reduce avian predation [18–21]. However, a quantitative model describing how best to choose coloration for a specific light distribution and animal shape has been missing. These earlier studies could not, therefore, answer the question of whether and how tightly a countershading pattern has to be related to the actual illumination of a scene to be effective. This empirical question, which has strong repercussions for our understanding of the role of countershading as visual camouflage, is the central question we address in the current work.

We recently described a mathematical model that draws knowledge from how light distribution, animal body geometry and orientation in space interact, to make predictions regarding what countershading patterns would be optimal for visual camouflage [5]. The modelling uses naturalistic lighting environments as described by the International Commission on Illumination [17]. Given an animal and a location, the model takes into account the interaction between the light in the (terrestrial) environment, which depends on weather (e.g. sunny or cloudy) and sun position (defined by latitude, time of the year, time of the day) and the geometry of the animal body, to deduce the optimal colour pattern to minimize shading information. This modelling shows, for example, that, all other conditions being equal, theoretically optimal colorations for a cloudy or for a sunny sky differ [5]. This can be simply understood from figure 1: the countershading pattern that counterbalances self-shadowing under a cloudy sky (figure 1*b*) has a shallower gradient than that for the stronger self-shadowing under a sunny light distribution (figure 1*c*).

We have recently used this model to demonstrate that the structural differences between cloudy and sunny weather affect predation by a population of wild birds [22]. We used our model to develop optimally shaded paper cylinders, containing a food bait, which were distributed in a UK woodland. Predation rate was measured on sunny and cloudy days, for cylinders countershaded optimally for both weather conditions. Predation was lower when the countershaded bait matched the optimal countershading pattern, providing the first evidence that fundamental differences in countershading can affect survival rates in countershaded prey.

In this study, we use a laboratory-based study with human observers to tackle the more specific question of how visibility changes with differences in countershading that are consistent with different lighting conditions. This question is important to determine the possible constraints on countershading camouflage and its interaction with animal behaviour. If countershading must be tightly matched to the light distribution to be maximally effective, an animal may have to restrict its activity to a specific environment to reduce predation risk to a tolerable level, which may have an opportunity cost. On the other hand, it may be possible to deploy a compromise countershading pattern that is cryptic for different illuminations, with a lower opportunity cost. In a study based on the same experimental setting, we found that whereas target orientation in space should be matched to the reference orientation, a reasonable range of deviation in orientation was still possible within which search performance was not affected [23].

To understand whether differences in countershading pattern have an impact on visibility, we assess visibility by measuring how quickly and accurately ovoid targets can be found amidst a cluttered background, whose elements have the same two-dimensional profile. We measure how performance changes for different levels of match between target body pattern and optimal pattern for a given light distribution. To do this we defined visual scenes for presentation on a computer monitor, containing a target object and several distractor items. The target objects were rendered 3D ellipsoids that had an optimal countershading pattern for one of the two light distributions, or were uniformly coloured; distractors had the same two-dimensional form (ellipses) but were rendered as folded ‘leaves’. In the real world, animals face a wide variety of lighting environments. Here, we chose to study two specific conditions, consistent with bright sunshine or with full cloud cover. These reflect two likely environmental conditions, from different regions of the ‘space’ of possible lighting conditions. The scenes were thus illuminated using two types of light: a diffuse light consistent with cloudy weather, and a more direct light, consistent with sunny weather. We then used a standard visual search paradigm [24–27] where observers searched for the target item.

We chose human participants as they can be considered a generalist predator, and a good model for studying visual camouflage [28–31]. Human observers were asked to find the target object among distractors on a computer monitor as quickly as possible. A low accuracy (target rarely found) and long detection time (for accurate responses, a long delay to find the target) are signatures of target survival, and, hence, of enhanced camouflage. The advantages of a laboratory setting using human participants over the predation experiment with wild birds [22] are twofold. First, it allows for full control of the experimental conditions. More specifically, we can create a perfect match between the optimal (i.e. the ‘best’ camouflage) pattern of reflectance on the target object and lighting and assess how visibility is altered with departure from optimality. Second, we can ask participants to engage in a task where they are specifically asked to search for a particular target, with no explicit training or learning required.

Using this experimental setting, we provide evidence that differences in countershading pattern, due to prevailing weather conditions, do contribute to making detection more difficult. We show that even a small deviation of shading pattern from the optimal pattern delivers a dramatic increase in visibility.

## 2. Material and methods

### 2.1. Calculation of optimal countershading

To build our stimuli and create scenes with realistic light distributions, we used the modelling described in [5]. This work provides a way to set up 3D scenes and render them using ray-tracing techniques with the software Radiance [32]. The 3D scenes can be illuminated by realistic light distributions as described by the International Commission on Illumination [17] in which ecologically relevant variables such as latitude, time of the day, time of the year and sky type (weather) can be specified. No cover was interposed between the scenes and the sky; thus we modelled the interaction between each target object and the light in a completely bare environment. This modelling allows us to mathematically predict the optimal reflectance on the body for visual camouflage (where reflectance is the proportion of the light impinging on the body that is reflected off its surface). Here, we used two types of illumination: cloudy, which provides diffuse illumination, and sunny, which gives a more direct illumination with a strong directional component in the direction of the sun (see [5] for further details). Once a scene is defined, our model [5] allows us to compute the amount of light (irradiance) falling upon each part of the body and, under the simplifying and common assumption that the reflectance of the body is Lambertian (i.e. it is matte rather than mirror-like [33,34]), compute the pattern of reflectance on the body that gives rise to the minimal shading for the lighting of the scene (we call this the optimal countershading pattern). In natural environments, most of the light comes from above. Optimal patterns of reflectance to deliver minimal shading are therefore countershaded [5].

### 2.2. Stimuli

**Figure 2.**
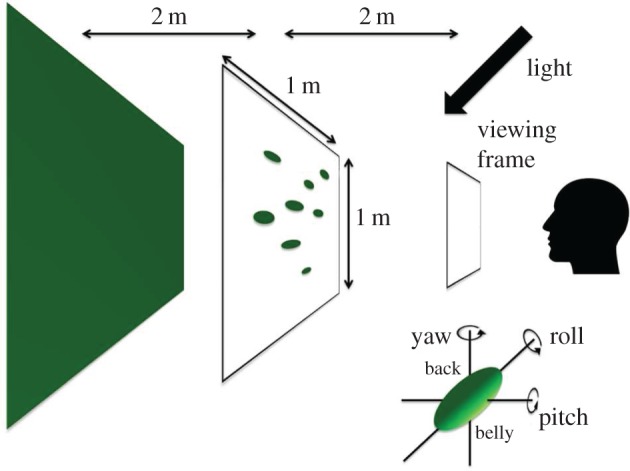
Three-dimensional arrangement of the scene featured in the search task (see text for details). Body orientation is described using pitch, yaw and roll, as shown in the inset.

**Figure 3.**
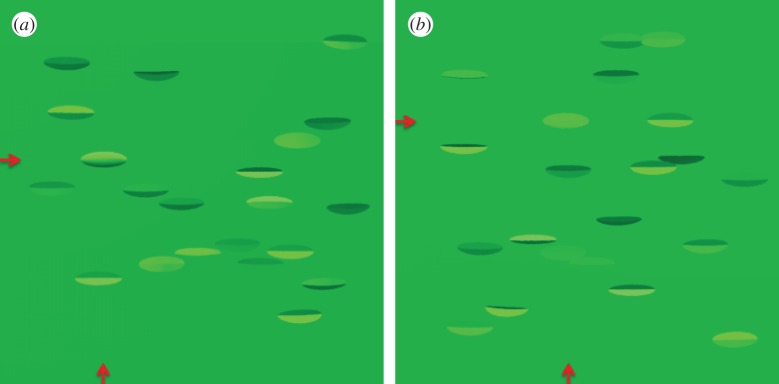
Illustration of the stimuli. (*a*) The target object (*x* and *y* location indicated by the red arrows for illustration here; arrows were not present in the experiments) has a uniform reflectance. (*b*) The target object has the optimal profile for the actual lighting of the scene (cloudy). More examples of the stimuli are shown in electronic supplementary material, figure S2.

3D scenes contained a uniform green backdrop, simulated to be 4 m from the observer. We created the target and distractors in such a way that the sole cue to target identity was shading. Target and distractors therefore had to have similar size, colours and, in particular, outline, so that the target could not be identified by its two-dimensional shape alone. The target was an ellipsoid, suggesting a caterpillar body. Distractors were built (as virtual ‘leaves’ within the computer program) from flat ellipses resulting from the projection of a target ellipsoid onto a plane parallel to its major axis and then folded along the minor axis (*x*) and curved along the major axis (*y*). The leaf fold was achieved by rotating each half of the leaf inwards across its major axis by 15°. Then curvature was added along the major axis by altering the (vertical) *z*-axis locations by *c* × cos(*y/l*), where *c* is a random number with a mean and standard deviation of one-sixth of the length of the major axis *l* (120 mm in the 3D model). These operations ensured that leaves could have had a wide range of surface normals, and therefore a wide range of brightness levels across the leaf surfaces. Each leaf was oriented so that its long axis was horizontal in order that its two-dimensional elliptical profile would be similar to that of the targets. This resulted in distractors typically having an almost two-tone appearance (for examples, see figure 3). We displayed a single target and either 20 or 40 distractor items distributed across the scene, with object centres in a fronto-parallel plane, simulated to be 2 m from the observer (figure 2). A large separation between this plane and the backdrop meant that no shadows were cast on the backdrop. To ensure that physical location carried no information regarding target location, the *y*--*z* locations, i.e. that in the fronto-parallel plane, of all the objects in the 3D scenes (both distractors and target) were drawn randomly from a uniform distribution. The location of the target was then chosen at random among these objects' locations. To rule out two items occupying the same location in virtual space, only scenes in which all the items' centres were separated from each other by at least 1.3 times the distractor length were used. The ellipsoidal target item was always horizontal, with the reference orientation pitch = roll = yaw = 0° (see figure 2, inset, for definition of pitch, roll and yaw). To ensure that orientation was not a cue to the identity of the target, the orientations in space of the distractors were constrained similarly; their long axis had the same orientation as that of the target item (pitch and yaw were fixed to 0°; roll was given random departure from the reference orientation and was normally distributed with mean = 0°, *σ *= 50°).

The scenes were rendered in Radiance [32] with two types of light distribution, a light distribution corresponding to a cloudy sky and a light distribution corresponding to a sunny sky. Radiance produces an accurate representation of chromaticities [35] and natural light distributions [17] in a scene. Because shading and cast shadows are accurately represented, we needed to avoid cast shadows cueing the participant to the location of the target. With the sun simulated to be above and behind the viewer (azimuth 270°, altitude 45°), the majority of shadows cast by items in the display were not cast upon items lower down within the arrangement, rather they were cast upon the distant backdrop outside the field of view. Nevertheless, due to the pseudorandom nature of the stimulus arrangement, occasional cast shadows were visible upon leaves and targets that were located lower down within the stimulus. These shadows would be as likely to be present in all experimental conditions, and, importantly, cast onto the target and distractors with the same probability, and therefore would not significantly influence performance. Figure 3 and electronic supplementary material, figure S2, provide examples of the rendered scenes.

Each target and distractor item occupied a visual angle of 1.27° × 0.4° (along the long axis and short axis of the body, respectively). The whole scene measured 10.58° by 10.58° of visual angle. On-screen chromaticities of the items were matched (in the CIE 1931 space, the mean chromaticities of the elements composing the stimuli were: target xyY = [0.14,0.52,19.91], with respective ranges [0.13,0.15], [0.50,0.52] and [13.42,34.41]; distractors xyY = [0.14,0.52,21.33], with respective ranges [0.13,0.15], [0.50,0.52] and [14.80,35.14]; background xyY = [0.14,0.52,21.56], with respective ranges [0.13,0.15], [0.50,0.52] and [14.92,35.15]). The images rendered in Radiance, which have a high dynamic range, were rescaled to adapt to the dynamic range of the screen in such a way that 99% of the pixels were below the maximum available value of 1. The brighter remaining values were clipped to 1. The resulting luminance of the items in the scenes ranged between 13 and 35 cd m^−2^.

### 2.3. Rendering weather-specific scenes

As explained above, optimal countershading (CS) patterns for reducing shading cues depend on the light distribution in a scene. Consequently, different light distributions deliver distinct optimal patterns. Scenes were rendered using one of two types of lighting: (i) sunny, in which the pattern of light is direct and shows a strong gradient between the direction of maximal illumination, and directions of minimal illumination, and (ii) cloudy, which is more diffuse and has a reduced gradient of illumination. Half of the scenes (60) were rendered with sunny lighting and half with cloudy lighting. The target object was alternatively endowed with a uniform reflectance pattern (referred to for the rest of the paper as ‘no CS’), with a countershaded pattern optimized for cloudy lighting (cloudy CS), or with a CS pattern optimized for sunny lighting (sunny CS). Targets with a CS pattern (i.e. sunny CS and cloudy CS) will be referred to as *camouflaged* in the remainder of the paper.

Three stimulus scenarios were possible: (i) the pattern of reflectance on the target body could be optimal for the actual light distribution of the scene; (ii) the pattern of reflectance could be optimal for a different light distribution; and (iii) a uniform coloured object (no CS), which would not be optimal for either of the light distributions. Figure 3 shows two examples of scenes used as stimuli in the experiment. The red arrows on *x*- and *y*-axes in the figure are for illustrative purposes only, and point towards the location of the target, which lies at the intersection of these two directions. In figure 3*a*, the target has a uniform reflectance and is rendered under cloudy conditions. The shading created by the pattern of illumination is therefore clearly discernible on the body, making it easy to detect. In figure 3*b*, the reflectance of the target is optimal for the actual lighting of the scene (i.e. cloudy CS under a diffuse illumination) and therefore has a uniform appearance, showing no shading information. It is harder to tell apart from the flat distractors.

### 2.4. Apparatus

Images were shown via a Wheatstone stereoscope, a device that allows the presentation of a different image to each eye, when presented side by side on a screen. For this experiment we did not exploit this utility. Instead, identical images were projected to each eye (we had planned a possible binocular disparity manipulation but this was not done here). Thus, only *monocular* cues to depth were presented, as when viewing a standard TV or computer screen. Both left and right stimuli were displayed on the same screen (Mitsubishi Diamondtron 22 inch CRT monitor, frame rate 120 frames per second, viewing distance between eye and screen surface 810 mm) using a Visage display system (Cambridge Research Systems Visage, CRS, Rochester, UK). Participants placed their chin upon a rest in a dark room. Their responses were recorded using a CRS RB-530 response box.

### 2.5. Training and procedure

We recorded both proportion of correct target detection and reaction time in a visual search experiment. For each scene, participant responses were divided into two stages. In a first stage, participants pressed a dial (Griffin Powermate; Griffin Technology, Nashville, TN, USA) as quickly as possible when they detected the target (we call this the reaction time). Participants were then presented with a scrambled version of the scene for one second. In a second stage, a monochromatic version of the scene then appeared on the screen, which only showed information about the location of target and distractors. Target and distractors were coloured in uniform grey and the background in black. One randomly chosen item was highlighted in red. Participants were asked to rotate the dial until the object in the scene they considered to be the target was highlighted. This two-stage procedure allowed us to measure response time to detect the target (response time, stage 1), independently of whether the chosen location was correct (accuracy, stage 2) [36]. This procedure allowed us to measure both detection times, and accuracy, for each target–weather combination, for each participant. Typically, we expected observers to respond more quickly when the target was not countershaded at all (figure 3*a*) or was suboptimally countershaded. We also expected pattern optimality to affect the second stage of the procedure, with no CS (figure 3*a*) or suboptimal CS being detected with more accuracy than optimal CS (figure 3*b*).

This procedure was applied for the two weather conditions (sunny, cloudy) and three target conditions (CS cloudy, CS sunny, no CS). We ran two sessions of 120 stimuli for each participant. The 6 possible crossed conditions for the pair *scene lighting *× *reflectance optimization lighting* were balanced (giving 20 scenes for each). The number of distractors (20 or 40) was evenly distributed across the conditions. Each session started with a training set of 10 stimuli, for which data were not recorded, followed by the 120 test stimuli. A total of nine participants (seven female) took part in the study; all were students of the University of St Andrews.

### 2.6. Statistical analysis

The dependence of search performance on divergence from optimal camouflage was analysed using generalized mixed models. ‘Participant identity’ was included in the models as a random effect. We used gamma distributions to model the distributions of reaction time [37] and binomial distributions (target found, target missed) to model proportion correct. In both experiments, the fixed effects were light distribution (two levels, cloudy or sunny), the pattern of reflectance on the target object (three levels: no CS, cloudy or sunny CS) and experimental session (two levels). We used the function *glmer* from the package *lme4* [38] in R (R v. 3.1.0) to fit the generalized mixed models. We used the Tukey procedure from the R package *multcomp* [39] to perform the multiple comparisons between predictor levels. All data and R code providing analysis and figures are available on the Open Science Framework (https://osf.io/c3n4s/) [40].

## 3. Results

**Figure 4.**
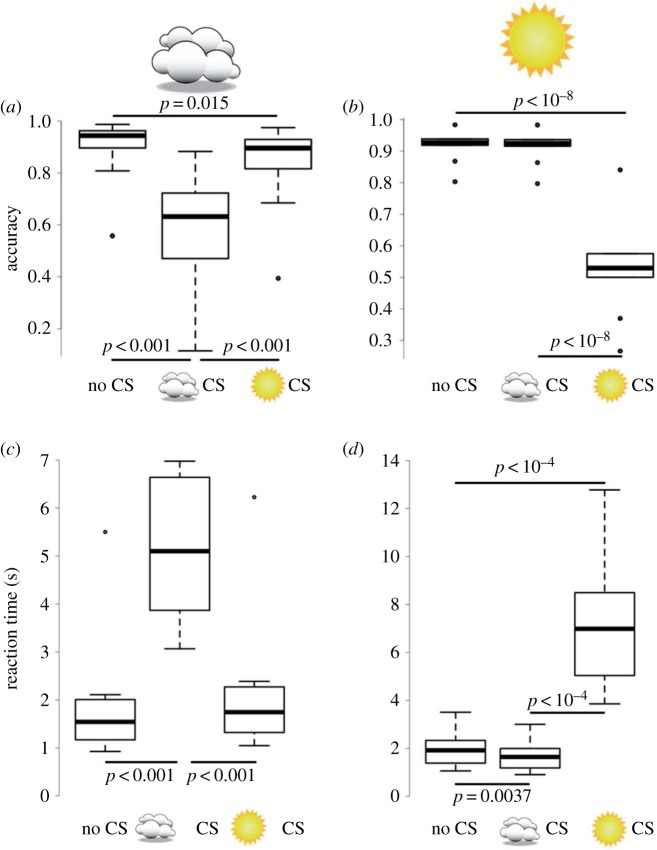
Results for human observer experiment. Detection accuracy, measured as proportion correct (*a*,*b*) and reaction time (*c*,*d*) as a function of agreement between scene lighting and reflectance pattern on the target object. The left column (*a*,*c*) corresponds to cloudy scenes and the right column (*b*,*d*) to sunny scenes. Reflectance patterns on the target object are represented by no logo (no CS), a logo depicting a cloud (cloudy CS) or a logo depicting the sun (sunny CS). Box plots display quartiles, with whiskers extending to the first point within 1.5 interquartile ranges of the box. Any points beyond the whiskers are plotted as asterisks. Horizontal bars show significant differences as determined by the Tukey adjusted test for pairwise comparisons between reflectance patterns (see electronic supplementary material, table S1, for details). Non-significant differences are not shown.

We measured detection accuracy and reaction time for the two scene types and three target types (figure 4). There was a decrease in accuracy (proportion correct) and a slowing of reaction times when the CS pattern matched the ‘weather’ of the scene in which it was presented. In cloudy weather, the cloudy CS objects were detected correctly less often (figure 4*a*; *χ*^2^ = 136.14, d.f. = 2, *p* < 10^−15^), and the reaction times were longer (figure 4*c*; *χ*^2^ = 199.28, d.f. = 2, *p* < 10^−15^) than they were for sunny CS and uniform coloration (electronic supplementary material, table S1A, dark grey cells below the diagonal). It also took significantly more time for participants to detect this pattern under cloudy light than the other light distributions (see electronic supplementary material, table S1A, for details of pairwise comparisons, light grey cells above the diagonal). Experimental session had no effect on proportion correct (*χ*^2^ = 2.54, *p* = 0.11), but had an effect on reaction time, with the target being detected significantly faster during the second session than during the first one (*z* = −5.64, *p* < 10^−7^).

For sunny weather, the sunny CS target was detected less often (figure 4*b*) and the reaction times were longer (figure 4*d*). Detection accuracy was significantly lower for sunny CS objects (electronic supplementary material, table S1B, dark grey cells) and reaction time was significantly reduced (electronic supplementary material, table S1B, light grey cells). Experimental session had an effect on proportion correct and reaction time, with the target being detected more accurately (*z* = 2.47, *p* = 0.014) and faster (*z* = −6.75, *p* < 10^−10^) in the second session.

Note that, although CS was much more effective when the CS pattern and weather were matched, in cloudy weather the sunny CS pattern provided improved crypsis over the uniform coloration, indicated by reduced detection accuracy (*z* = −2.78, *p* = 0.015). The difference in reaction times was not significant (*z* = 2.30, *p* = 0.056). This pattern was not as evident for the cloudy CS pattern under sunny weather conditions; here the reaction time was significantly different from the uniform pattern (*z* = −3.21, *p* = 0.0037), but the accuracy was not (*z* = −0.14, *p* = 0.99). What this does show, however, is that CS, even when far from optimal, shows a trend towards delivering a small survival advantage over no CS. We did not find any specific difference when excluding incorrect trials from the analysis (see electronic supplementary material, figure S1).

## 4. Discussion

Our aim was to explore the extent to which CS affected the ability to detect target objects, for lighting conditions where the target was optimized for those conditions, optimized for other lighting conditions, or uniform. We found that people were less accurate and slower to find an ellipsoid 3D target ‘caterpillar’, displayed among distractor objects in the form of flat ‘leaves’, when the target object was endowed with a pattern of optimal countershading which minimized self-shadowing by exactly counteracting the current light environment. Crucially, patterns optimized for other lighting conditions, or not optimized at all (uniform patterns), made the target object significantly more visible. The effect was large: there was a 2–4 times increase in reaction time and overall around a 50% decrease in correct detection between the scenes in which the target had the optimal colour pattern and the scenes in which the pattern was suboptimal or uniform. A good match between the actual illumination of the scene and the reflectance pattern on the target object therefore caused a dramatic reduction in detectability. These findings show how scene changes that simulate realistic changes in the natural world can drastically affect even targets that have some countershading camouflage. They support the hypothesis that visual camouflage may have been one of the driving forces for the evolution of the countershading pattern that is so common in nature [15,41,42]. They are also consistent with the previous observation that the countershading patterns of species living closer to the equator or in open lighting environments have a stronger gradient [7]. Below, we explore the implications of our results for the following questions: (i) How ‘good’ does countershading have to be to be an effective foil against a predator? (ii) Does the countershading eliminate a cue to 3D vision? Finally, we reflect on the limitations of this study in terms of the ‘naturalness’ of the virtual environment used.

### 4.1. Is suboptimal countershading ‘good enough’ as camouflage?

Most animals are active throughout the full day and restricting their activity to areas with a homogeneous illumination may have important opportunity costs. Furthermore, in most climates illumination can vary frequently between diffuse and direct. The question of finding a trade-off, optimal countershading profile is therefore crucial for understanding how countershading works as camouflage. To address the issue of finding the best compromise camouflage, suitable for a wide range of climatic conditions, we first need to know how sensitive predators' visual systems are to differences in CS pattern.

Perhaps not surprisingly, we found that, having no countershading (i.e. having uniform coloration) damaged crypsis (i.e. increased detection accuracy and reduced reaction time). Importantly, having suboptimal countershading (i.e. cloudy CS under a sunny sky) was almost as damaging for crypsis as having no countershading at all. This result may partially reflect the difference between the two lighting distributions that we chose: the CIE sunny sky is maximally directional, whereas the cloudy sky is very diffuse (albeit with a strong light-from-above gradient). In the real world, a foraging animal may be exposed to a wide range of illumination gradients, and thus one might expect evolution of a compromise suboptimal pattern that could provide partial crypsis. Although we found there to be a cost of having the wrong CS pattern, one can still argue that having either pattern will be better when faced with a range of lighting conditions than having no countershading whatsoever. For example, if the illumination were sunny 70% of the time and cloudy 30% of the time, then having a sunny CS pattern would result in the best overall survival rate, but those animals with the cloudy CS would still survive better than those with a uniform CS pattern. Only further studies with a more gradual variation in illumination might reveal what pattern of camouflage might be the best overall compromise between countershading for sunny and diffuse illumination [6,7,23].

### 4.2. Do our data inform on whether countershading is ‘designed’ to remove a three-dimensional cue?

We found a small asymmetry in the efficiency of the suboptimal CS in the ‘wrong’ light environment. Cloudy CS was no different than uniform coloration under sunny illumination. Although under cloudy illumination sunny CS appears to offer a small advantage over uniform coloration, the difference in reaction times was not significant (*z* = 2.30, *p* = 0.056). This pattern of results can be understood in reference to shape from shading cues (e.g. [43]), whose use could contribute to target detection. A cloudy CS ellipsoid presents a pattern of radiation consistent with a convex shape (light at the top and dark at the bottom), while a sunny CS ellipsoid under cloudy illumination will have a radiation pattern consistent with a concave shape (dark at the top and light at the bottom). In the context of our study, the strongest countershading pattern, namely the one that never delivers a convex appearance to the objects, could provide a survival advantage overall.

### 4.3. Comparing the effect of countershading on birds and humans

Our results can also be contrasted with those obtained in a companion study of avian predation in the field [22]. In this study, the same modelling was used to design ‘optimal’ countershading patterns, which were applied to real objects. Model baits containing these patterns were placed on bushes and the predation rate by a population of local wild birds, after a fixed time, was measured. We found that countershading favours camouflage overall, and, importantly, that optimally effective countershading strongly depends on lighting conditions, in agreement with the present study in the laboratory. In the field study, however, the differences in predation between optimal and suboptimal profiles were strongly reduced, compared with our laboratory-based data presented here. In the laboratory, we were able to specifically isolate the countershading cue, and variation of lighting treatment on the target was the only variable tested, whereas several intertwined factors that we could evaluate were inevitably at play in the experiment run in a natural, complex environment.

Of course, there is also the issue of comparing performance across species. The earliest accounts of countershading [3,14] assumed that reduced visibility stems from an improved background matching when seen from above or from below, or a reduction in self-shadowing. Humans are very sensitive to shape-from-shading (e.g. [43–47]). Other species have also been shown to be sensitive to shading information. Chimpanzees (*Pan troglodytes*) are able to use shading patterns in segregation tasks [48]. Pigeons (*Columba livia domestica*) [49] and starlings (*Sturnus vulgaris*) [50] can discriminate convex from concave shapes. Using human observers for testing camouflage in a visual search task offers several advantages. Humans have a complex, evolved visual system that is well studied. In particular, how humans perceive shaded objects is well understood. Humans also are generalist predators. Importantly, humans can be given complex instructions. In our study, we asked participants to locate, as quickly and as accurately as possible, the target which was referred to as a volume. We know that shading elicits a strong perception of three-dimensionality in humans [43,44,46,47], but as we point out above, our experiments do not directly test whether it is the loss of a 3D percept that slows search. In other animals, Qadri *et al.* [50] have recently shown that starlings can reliably discriminate between realistically rendered images of concave and convex shapes. In that study, starlings' discrimination performance remained unaffected by potentially confounding effects such as viewing perspective, contrast, colour of the illuminant or specularities. The authors concluded that relative shading is likely to provide a strong feature for 3D shape perception in birds. Another feature, contour, is inherent to the design of this kind of experiment, however (see [49] for a discussion in a similar experiment with pigeons), and the hypothesis that starlings, and pigeons, use contours as a cue to discriminate convex from concave cannot yet be ruled out. Like those of others [49–51], and despite using human observers, our experiment too is inconclusive regarding the strategy employed in the search task.

### 4.4. Environment complexity and study limitations

Several parameters underlying the interaction between body shape, orientation and light distribution, such as the impact of solar elevation for example, are not captured by the simple scenes used in this study. Moreover, natural environments vary over time, with substantial variations at different time scales in the light distribution, whether induced by abrupt changes of weather or cyclic variations along day and year. In this study, we wanted to single out and examine the putative main impact of the countershading pattern on visibility, namely that of decreasing visibility by reducing the shading on the body. We chose to compare two quite different environments that would deliver very different optimal countershading patterns. The extent to which these results can be extended to more complex light--target arrangements, including the ‘dappled shade’ complex environments that are probably more realistic, is hard to predict, and is beyond the scope of this study. In general, adding more noise to both target and to distractor items increases the overall heterogeneity of the scene and results in slower target detection [52]. In a related study, we have explored the effects of object rotation around three different axes and show that quite subtle differences in body orientation can have a marked effect on target detection [23]. The latter work compares with the current study in the sense that it uses the same setting and experimental design, and importantly can isolate the effect of compatibility between countershading pattern and light distribution as the only variable tested. On the other hand, the current study reproduces, in a controlled environment and with humans, i.e. exemplar predators whose visual system is well understood, the illumination-dependent effect of countershading on detectability found in the wild [22], which allows us to speculate on possible perceptual mechanisms underlying the effect. Taken together, the study of the effects of body rotation on visibility [23], the present study and the study of avian predation in the field [22] provide converging support for the benefits of removing shading cues on the body as predicted in [6].

## 5. Conclusion

In a simulated 3D environment with realistic light distributions, human visual search was less effective, i.e. less accurate and slower, when the target object had a countershading that was optimal for the actual lighting of the scene, compared with both non-camouflaged and non-optimally camouflaged patterns. From many studies, we know that our visual systems are able to use reliably shading cues as a proxy to 3D shape. While we cannot be sure here that observers used shading as a 3D cue to detect the target, we have shown clearly that the different reflectance gradients delivered by lighting environments that emulate different ‘weather’ make a large difference to our ability to perceive countershaded targets.

## Supplementary Material

data_horizontal_leaves.txt

## Data accessibility

All data and R code providing analysis and figures are available on the Open Science Framework (https://osf.io/c3n4s/) [40].
